# Isolation, Identification, and Antitumor Activities of Glucosinolates and Isothiocyanates in Chinese Cabbage Seeds

**DOI:** 10.3390/foods14162808

**Published:** 2025-08-13

**Authors:** Bei Zhou, Ying Liu, Xi Feng, Qian Liu, Salam A. Ibrahim, Wen Huang

**Affiliations:** 1 College of Food Science and Technology, Wuhan Business University, Wuhan 430056, China; zhoubei_wbu@163.com; 2 College of Food Science and Technology, Huazhong Agricultural University, Wuhan 430070, China; yingliu@mail.hzau.edu.cn (Y.L.); liuqian202508@163.com (Q.L.); 3 Department of Nutrition, Food Science and Packaging, San Jose State University, San Jose, CA 95192, USA; xi.feng@sjsu.edu; 4 Department of Family and Consumer Sciences, North Carolina A & T State University, 171 Carver Hall, Greensboro, NC 27411, USA; ibrah001@ncau.edu

**Keywords:** Chinese cabbage seed, glucosinolate, Isothiocyanate, UHPLC-Q-TOF-MS, identification, antitumor activity

## Abstract

Isothiocyanates (ITCs), which are derivatives of glucosinolates (GSLs) from *Brassica* plants, have been investigated as anticancer agents. An extensively studied anticancer ITC is sulforaphane, which is found in low amounts in Chinese cabbage. We aim to investigate the types and content of GSLs (precursors of ITCs with anticancer activity) in Chinese cabbage seeds. GSLs from Chinese cabbage seeds were isolated and purified using acidic Al_2_O_3_ column chromatography and preparative HPLC. GSL and ITC profiles were further identified using UHPLC-Q-TOF-MS. The antitumor activities of ITC (produced by exogenous enzymatic hydrolysis of GSLs) were evaluated in vitro. Seventeen GSLs and seven ITCs were identified, and the dominant GSLs were gluconapin, glucobrassicanapin, and progoitrin in Chinese cabbage seeds. High-purity gluconapin (>99%) was purified. The ITCs showed synergistic-, dose-, and time-dependent effects on the inhibition of HepG2 cells, and the key ITCs were 3-butenyl ITC, sulforaphane, and 2-phenylethyl ITC. The corresponding parent GSLs were gluconapin, glucoraphanin, and gluconasturtiin, respectively. 3-Butenyl ITC could significantly induce HepG2 cell proliferation (IC50 = 89.44 μg/mL) and apoptosis (*p* < 0.05). Our results suggested that Chinese cabbage seed could be a valuable source of natural antitumor ingredients.

## 1. Introduction

Glucosinolates (GSLs), sulfur-rich secondary metabolites, are particularly abundant in the genus *Brassica* of the *Brassicaceae* family [1,2]. Based on their precursor amino acids, glucosinolates can be classified into aliphatic, aromatic, and indolic glucosinolates [1]. The hydrolysis of GSLs by myrosinase in plants [2] yields a range of breakdown products, including isothiocyanates (ITCs), nitriles, epithionitriles, thiocyanates, and oxazolidine-2-thiones. These compounds, especially isothiocyanates (pH 5–7), have anticancer, antimicrobial, and antifungal effects in vitro and in vivo, animal, human, and epidemiological studies [1,2,3].

An increasing amount of data indicates that consuming *Brassica* vegetables regularly decreases the risk of breast cancers [4], lung cancers [5], prostate cancers [6], stomach cancers [7], colorectal cancers [8], pancreatic cancers [9], and bladder cancers [10]. Isothiocyanates, which are derivatives of glucosinolates from *Brassica* plants, include sulforaphane (SFN), phenethyl isothiocyanate, allyl isothiocyanate, benzyl isothiocyanates, and erucin [10,11,12,13] and have been investigated as anticancer agents and relevant evidence has validated their efficacy [5,6,7,9].

Chinese cabbage (*Brassica rapa* L. ssp. *pekinensis*) is a widely grown vegetable [14]. A previous study revealed that the main glucosinolates in Chinese cabbage were gluconapin (NAP), progoitrin (PRO), and glucobrassicanapin (GBA) [15]. However, the concentration of glucoraphanin (GRA) in Chinese cabbage is lower than in broccoli, which has an anticancer effect due to its metabolite sulforaphane [1]. Furthermore, it remains to be determined if other isothiocyanates exhibit anticancer activity in Chinese cabbage.

In this study, we aimed to isolate and purify glucosinolates from Chinese cabbage seeds, and then implement in vitro cellular assays to investigate the anticancer activity of ITCs (enzymatic digestion by exogenous enzymes) and derivatives of different GSLs fractions. Ultrahigh-performance liquid chromatography–quadrupole time of flight mass spectrometry (UHPLC-Q-TOF-MS) was used to identify glucosinolates and isothiocyanates. MTT assay, AO/EB staining, and apoptosis were used to assess the anticancer activity of ITCs. We hypothesize that beyond known reported cases, there will be new active glucosinolate derivatives with antitumor activity.

## 2. Materials and Methods

### 2.1. Materials

Chinese cabbage seed, namely Zaoshu-5 (Validation code: GDP Cc (2018) 330928), was purchased from ShenLu Seed Co., Ltd. (Wenzhou, China).

The chemicals used were HPLC grade. Standards of 2-propenyl glucosinolate (sinigrin, SIN) were >98% pure, purchased from YuanYe Bio-Technology Co., Ltd. (Shanghai, China). Myrosinase, from *Sinapis alba* (white mustard) seed, was purchased from Sigma-Aldrich (St. Louis, MO, USA). Palladium Chloride and N-acetyl-cysteine (99% pure) were purchased from YuanYe Bio-Technology Co., Ltd. (Shanghai, China). Human hepatocellular carcinoma (HepG2) cell lines, human colon carcinoma (HT-29) cell lines, human osteosarcoma (SW1353) cell lines, human prostate cancer (DU145) cell lines, and human cervical carcinoma (Hela) cell lines were purchased from Chinese Academy of Sciences (Shanghai, China). MEM Basal Medium, DMEM Basal Medium, and RPMI-1640 Basal Medium were purchased from Gibco (San Francisco, CA, USA). Fetal Bovine Serum (FBS) was purchased from Gibco (San Francisco, CA, USA). Penicillin–Streptomycin Solution (Dual Antibiotic) was purchased from genview (Beijing, China). Trypsin was purchased from GENOM Bio. Co., Ltd. (Hangzhou, China). Cell Freezing Medium was purchased from Biosharp life sciences (Hefei, China). Thiazolyl Blue Tetrazolium Bromide (MTT) was purchased from biofroxx (Einhausen, Germany).

### 2.2. Glucosinolate Extraction

Glucosinolate extraction followed established protocols with minor adjustments [16]. Chinese cabbage seeds were ground into powder in a mortar and the powder (5 g) was immersed in a boiling water bath for 5 min to inactivate the endogenous myrosinase. Lipid removal was achieved by stirring seed meal with 30 mL petroleum ether for 3 h [17]. The defatted material underwent sequential extraction: heating with 150 mL 70% (*v*/*v*) methanol at 70 °C for 15 min; subsequent sonication (600 W) at 25 °C for 15 min. The combined extract was filtered, concentrated under reduced pressure to remove methanol, reconstituted in ultrapure water, and lyophilized. Processed extracts were maintained at −20 °C prior to analysis. All procedures were performed in triplicate (n = 3).

### 2.3. Isolation and Purification of Glucosinolates

Acidic alumina (Al_2_O_3_) column chromatography was performed to improve the purity of crude glucosinolate extracts. The extract (120 mg) was dissolved in distilled water (4 mL) for elution with 0.1 M KNO_3_ solution at the flow rate of 0.6 mL/min by acidic Al_2_O_3_ column chromatography (16 × 20 mm). Fractions were collected and colorimetrically screened using PdCl_2_ colorimetry. The elution solution was evaporated and then dissolved in methanol for the removal of KNO_3_. Afterward, the filtrate was resolubilized in ultrapure water and freeze-dried. Total glucosinolate content in crude extracts and purified fractions of Chinese cabbage seeds were determined using the palladium chloride colorimetric method. A total of 40 μL of the sample solution (1 mg/mL) was added to a 96-well microplate, followed by the sequential addition of 80 μL of 0.15% sodium carboxymethyl cellulose solution and 80 μL of 0.04 mol/L palladium chloride solution. After thorough mixing, the reaction proceeded in the dark at room temperature for 2 h. Absorbance was measured at 540 nm. Sinigrin (2-propenyl glucosinolate) was used as the standard for quantitative analysis. Standard solutions at varying concentrations (0.1, 0.2, 0.3, 0.4, and 0.5 mg/mL) were prepared and processed following the same procedure as the samples. The standard curve was generated by plotting glucosinolate concentration (x-axis) against absorbance (y-axis). The regression equation was “y = 0.0603x + 0.0104 (R^2^ = 0.9991)”.

Waters 2545B preparative HPLC system (Waters, Milford, MA, USA) was utilised to isolate the crude glucosinolate extracts into different fractions. The sample (10 mg/mL) was analyzed on a SHIMADZU shim-pack GIST C18-AQ column (20 × 250 mm, 5 μm, Shimadzu, Japan) with the column temperature at 25 °C. Ultrapure water and methanol were used as mobile phases A and B, respectively. The gradient program was carried out as follows: 0.0 min, 90% A and 10% B; 30.0 min, 60% A and 40% B; 35.0 min, 100% B; 50.0 min, 100% B; 52.0 min, 90% A and 10% B; and 55.0 min, 90% A and 10% B. The flow rate was set at 5 mL/min. The injection volume was 2 mL, and the ultraviolet absorbance optical detector was set at 229 nm for analysis.

### 2.4. Degradation of Glucosinolates and Derivatization of ITCs with NAC

For the derivatization of the ITCs, the protocol previously described by Andini, Araya-Cloutier, Sanders, and Vincken was followed [18]. A stock solution of NAC (100 mM) was freshly prepared in a phosphate buffer (PBS) (pH 7.0, 0.1 M). Seed extracts (10 mg/mL, 0.5 mL), myrosinase (0.05 U), PBS (0.1 M, pH 7.0, 0.5 mL), and NAC (0.75 mL) were incubated at 50 °C for 4 h. Afterward, isopropyl alcohol (IPA) was added to a concentration of 25% (*v*/*v*). The samples were chilled at room temperature. Then the solution was passed through a 0.22 μm polyethersulfone filter and analyzed by UHPLC-Q-TOF-MS.

### 2.5. Identification and Characterization of NAC-ITCs

The analysis of NAC-ITCs was performed using UHPLC-QTOF-MS. This system was equipped with a Waters ACQUITY UHPLC BEH C18 column (2.1 × 100 mm, 1.7 μm) (Milford, MA, USA). Acidified water (0.1% formic acid, *v*/*v*) and acetonitrile (0.1% formic acid, *v*/*v*) were used as mobile phases A and B, respectively. The gradient program was carried out as follows: 0.0–6.7 min, 95% A and 5% B; 12.5 min, 92% A and 8% B; 24.2 min, 84% A and 16% B; 41.8 min, 60% A and 40% B; 43.5 min, 100% B; 50.5 min, 100% B; 52 min, 95% A and 5% B; and 59 min, 95% A and 5% B. The flow rate was set at 0.30 mL/min throughout the gradient. The injection volume was 1 μL, and the column temperature was maintained at 25 °C [18]. Parameters for analysis were set in a positive ion mode, with spectra acquired over a mass range from *m*/*z* 80 to 1000. The optimal values of the ESI-MS parameters were capillary voltage, 3 kV; source temperature, 135 °C; desolvation temperature, 300 °C; cone gas, 0.8 L/min; drying gas flow, 10.0 L/min. Moreover, automatic MS^E^ experiments were performed using nitrogen as the collision gas, with the low collision energy at 6.00 eV and the high collision energy ranging from 20.00 eV to 30.00 eV [18]. Sodium adducts [M + Na]+ and N-acetyl-L-cysteine [NAC + H]+ (*m*/*z* 164) were the other most abundant fragment ions for NAC-ITCs in the positive ion mode [19].

### 2.6. Identification and Characterization of Glucosinolates

The stock solutions (10 mg/mL) of crude glucosinolate extracts and purified fractions from Chinese cabbage seeds were thawed at 4 °C in a refrigerator. These were diluted with ultrapure water to a concentration of 200 μg/mL. An appropriate volume of sinigrin (2-propenyl glucosinolate) reference standard stock solution (2 mg/mL) was added to achieve a final concentration of 10 μg/mL. The test samples were filtered through a 0.22 μm nylon microporous filter, and the filtrate was injected for the qualitative and quantitative analysis of glucosinolates.

Chromatographic separation was achieved using a Waters ACQUITY UHPLC H-Class system (Milford, MA, USA) configured with a BEH C18 column (2.1 × 100 mm, 1.7 μm). The mobile phase consisted of eluent A, 0.1% (*v*/*v*) formic acid in water; and eluent B, 0.1% (*v*/*v*) formic acid in acetonitrile. A multistep gradient elution profile was implemented: 0.0 min, 95% A and 5% B; 6.0 min, 60% A and 40% B; 6.5 min, 100% B; 8.0 min, 100% B; 8.1 min, 95% A and 5% B; and 10.0 min, 95% A and 5% B. The flow rate was set at 0.40 mL/min. The injection volume was 1 μL, and the column temperature was maintained at 40 °C [16].

The UHPLC output was interfaced with a Vion IMS-Q-TOF mass spectrometer (Waters, Milford, MA, USA) featuring an electrospray ionization source (ESI). Data acquisition occurred during negative ionization mode scanning ‘*m*/*z*’ 80–1000. The optimal values of the ESI-MS parameters were capillary voltage, 3.5 kV; source temperature, 135 °C; desolvation temperature, 550 °C; cone gas, 0.8 L/min; and drying gas flow, 10.0 L/min. Moreover, automatic MS^E^ experiments were performed using nitrogen as the collision gas, with the low collision energy at 6.00 eV and the high collision energy ranging from 20.00 eV to 35.00 eV [16]. The MS data acquisition was processed with UNIFI software ver. 3.1 (Waters, Milford, MA, USA) to identify and quantify the glucosinolates. Instrument calibration was performed using real-time external mass calibration technology (Waters LockSpray ™ system with dual-spray ESI). Based on the cleavage pattern of glucosinolates in the negative ion mode of the ESI. The sulfur atom in the sulfonate aldoxime group was cleaved with the adjacent carbon atom and was rearranged with hydrogen atoms to produce the characteristic ions with *m*/*z* 274.9895, 259.0124, 241.0018, and 96.9596. The concentration of each glucosinolate was calculated by determining the relative values of each area with respect to the area of the internal standard (sinigrin, 10 μg/mL).

### 2.7. Cell Culture

HepG2 cells were cultured in MEM medium; HT29 cells were cultured in McCoy’s 5 A medium; SW1353 cells and Hela cells were cultured in DMEM medium; and DU145 cells were grown in RPMI-1640 medium. The medium for all cell lines was supplemented with 10% fetal bovine serum (FBS) and 1% antibiotic (containing 100 U/mL penicillin and 100 μg/mL streptomycin). Cell culture was performed at 37 °C in a 5% CO_2_ atmosphere (Thermo Scientific, Waltham, MA, USA). The cell culture medium was replaced every 2 days.

### 2.8. Cell Subculture and Counting

When cells reached 80–90% confluence in the culture flask, the old medium was aspirated. Cells were washed gently 1–2 times with sterile PBS. Subsequently, 1 mL of trypsin solution was added to the flask. The flask was swirled thoroughly for 30 s to ensure complete coverage of the cell monolayer with trypsin, after which the trypsin solution was aspirated. The flask was then placed in a 37 °C incubator for brief dry digestion (trypsin exposure without solution). Cell morphology was monitored under a microscope. Upon the observation of cell rounding and detachment, digestion was immediately stopped by adding complete medium. Cells were gently resuspended by swirling the flask or by gentle pipetting with a pipette. Once a uniform single-cell suspension was achieved, cells were subcultured (passaged) at the desired ratio according to the requirements of subsequent experiments.

For cell counting: Cells collected during subculture (or as required) were resuspended in culture medium by gentle pipetting to generate a single-cell suspension. A small drop of the cell suspension was placed onto a clean hemocytometer (counting chamber). Excess suspension and air bubbles were carefully removed, and a coverslip was applied. Cells within the large squares were visualized under a low-power microscope objective and counted.

Cell density (cells/mL) was calculated using the following formula:

Cell Density = (Mean number of cells per large square) × Dilution Factor × 10^4^ cells/mL

### 2.9. Sample Preparation for In Vitro Cellular Assays

Crude extracts, purified products, and collected fractions (10 mg/mL) were treated for 3 h at 37 °C with myrosinase (0.05 U), respectively. The samples were then diluted to the desired concentration with MEM medium and passed through a 0.22 μm syringe filter.

### 2.10. Antitumor Activity In Vitro

MTT assay was used to investigate the effect of ITCs on the proliferative activity of HepG2 cells. Sterile MTT powder was weighed in a biosafety cabinet and dissolved in PBS to prepare a 5 mg/mL stock solution. After complete dissolution, the solution was sterilized by filtration through a 0.22 µm aqueous sterile filter within the cabinet and stored at −20 °C until use. Prior to use, the stock solution was diluted 10-fold with basal medium (0.5 mg/mL). All preparation steps were performed under light-protected conditions. Before treatment, all cells were grown individually in 96-well plates at a density of 2 × 10^4^ cells/well for 24 h. After removing the medium, each well received 200 μL of varied concentrations of ITCs (obtained from Section 2.9) and was incubated at 37 °C for 24, 48, or 72 h. After discarding the medium, 150 μL MTT (0.5 mg/mL) was added to the wells and incubated at 37 °C for 4 h. The MTT was thrown away and replaced by adding DMSO (200 μL per well) and mixed for 10 min before measuring absorbance (optical density values, OD) at 490 nm in a microplate spectrophotometer. All groups were conducted in sextuplicate (n = 6).

Cell viability was calculated based on optical density (OD) values using the following formula:

Cell Viability (%) = [(OD__Test_ − OD__Blank_)/(OD__Normal Control_ − OD__Blank_)] × 100

where

OD__Test_ = Optical Density of the test group (treated cells);

OD__Blank_ = Optical Density of the blank control group (medium only, no cells);

OD__Normal Control_ = Optical Density of the normal control group (untreated cells).

The relationship between drug concentration and cell viability was analyzed using nonlinear regression curve fitting (Dose–response—Inhibition model) in GraphPad Prism ver. 7.0 (GraphPad, San Diego, CA, USA) to calculate the half-maximal inhibitory concentration (IC50).

### 2.11. AO/EB Staining

Complete medium (3 mL) was added to sterile glass bottom culture dishes (35 mm diameter, Sorfa, Huzhou, China). The dishes were placed in a 37 °C, 5% CO_2_ incubator for 15 min to pre-equilibrate the medium. HepG2 cells were collected and resuspended according to the standard subculture protocol. The cell density was adjusted to (1–2) × 10^5^ viable cells/mL. The pre-equilibrated dishes were removed from the incubator. The medium was carefully aspirated. Then, 500 μL of the cell suspension was evenly seeded onto the glass bottom surface of each dish. Following seeding, the dishes were returned to the 37 °C, 5% CO_2_ incubator and cultured for 2 h to allow cell attachment. After the 2 h attachment period, an additional 2–3 mL of complete medium was gently added to each dish. The cells were then cultured for a further 24 h.

Then the cells were treated with 2 mL of different concentrations of ITC-C3 (50 μg/mL, 100 μg/mL, and 200 μg/mL) for 48 h, each group with 3 replications. Following PBS washing, cells were stained with 50 μL AO/EB solution (AO/EB Double Stain Kit, Beijing Leagene Biotechnology Co., Ltd., Beijing, China) for 5–15 min at room temperature, followed by another PBS wash. The stained cells were photographed using a laser scanning confocal microscope (FV3000, OLYMPUS, Tokyo, Japan). Acridine Orange (AO) fluorescence: excitation wavelength = 488 nm; emission wavelength = 520 nm. Ethidium Bromide (EB) fluorescence: excitation wavelength = 543 nm; emission wavelength = 590 nm.

### 2.12. Apoptosis Assay

HepG2 cells were plated in a 6-well plate at a density of 2 × 10^5^ cells/mL, incubated for 24 h, and treated with 2 mL of different concentrations of ITC-C3 (50 μg/mL, 100 μg/mL, and 200 μg/mL) for 48 h, each group with 3 replications. The supernatant and cells were collected after being centrifuged at 1000 r/min for 3 min. Cells were washed with ice-cold PBS and gently resuspended by pipetting. The cell suspension was centrifuged at 1000 r/min for 3 min. The supernatant was discarded. The cell pellet was resuspended in an appropriate volume of 1× Binding Buffer. Cell concentration was determined microscopically and adjusted to 1 × 10^6^ viable cells/mL using 1× Binding Buffer. Next, 100 µL of the cell suspension (containing 1 × 10^5^ cells) was transferred to a 1.5 mL microcentrifuge tube, and the apoptosis of cells was estimated using the Annexin V-FITC/PI apoptosis kit (Multi Sciences (Lianke) Biotech, Co., Ltd., Hangzhou, China). Then, 5 µL of Annexin V-FITC and 5 µL of Propidium Iodide (PI) staining solutions were added to the cell suspension in each tube. Tubes were gently mixed and incubated for 15 min at RT in the dark. Following this, 400 µL of 1× Binding Buffer was added to each tube to resuspend the cells. Stained samples were analyzed using flow cytometry (Cytoflex-LX, Beckman, Brea, CA, USA) within 1 h of completing the staining procedure. Annexin V-FITC: detected in the FITC channel (excitation [Ex] = 488 nm; emission [Em] = 530 nm). Propidium Iodide (PI): detected in the PI/PE-Texas Red channel (Ex = 535 nm; Em = 615 nm).

### 2.13. Statistical Analysis

The results were presented as the mean ± standard deviation (SD). The data were processed using the IBM SPSS ^®^ ver. 19.0 (SPSS, Chicago, IL, USA). One-way ANOVA followed by LSD and Tamhane T2’s multiple-range test was used to evaluate the significant differences (*p* < 0.05). The software Origin ver. 9.0 (OriginLab Corporation, Northampton, MA, USA) and Graphpad Prism ver. 7.0 (GraphPad, San Diego, CA, USA) were used for graphing.

## 3. Results and Discussion

### 3.1. Purification and Identification of Glucosinolates

The crude GSL extracts from Chinese cabbage seeds were isolated and purified using an acidic alumina chromatography column eluted by 0.1 M KNO_3_ to obtain purer glucosinolate extracts. The elution solution (7–21 tubes) was collected, and the purity of the fraction CCG-S was 67.1%, which was 1.13 times higher than crude extracts of glucosinolates (CCG-CE) (*p* < 0.05). On the other hand, the crude GSL extracts were isolated and purified using preparative liquid chromatography. As shown in Figure S1A, seven fractions (C1-C7) were collected. The purities of CCG-(C1-C7) are shown in Figure S1B. Among all of the collected fractions, CCG-C3, CCG-C4, and CCG-C5 had higher purities, with 99.6%, 71.3%, and 64.4%, respectively. The research on the pharmacological activity of glucosinolates and associated metabolites has been severely hindered by the absence of easily available analytical standards [20]. Liquid–liquid partitioning and conventional column chromatography were typically utilized for the purification of glucosinolate crude extracts [21]. Glucosinolates could be significantly purified using column chromatography given that they were electronegative due to their sulfate groups [20]. Based on the differences in R-side chain groups, glucosinolates could be further isolated by utilizing stationary phases with various affinities and mobile phases [22,23]. Preparative reversed-phase liquid chromatography has been the most common method for the isolation of intact monomeric glucosinolates [23]. In our study, the purity of CCG-C3 reached 99.56%, demonstrating that preparative liquid chromatography was an effective approach for isolating glucosinolates.

The glucosinolate composition of the crude extracts and purified fractions were identified and quantified using UHPLC-Q-TOF-MS. The results indicated that a total of 17 glucosinolates were detected. The mass spectrometry data of the identified glucosinolates are shown in Table 1. Of these seventeen glucosinolates, ten belonged to aliphatic glucosinolates (NAP, GBA, PRO, GJP, GNF, GRS, GER, GBT, GRA, GAS), four belonged to indolic glucosinolates (GBS, 4HGBS, 4MGBS, NGBS), and three were aromatic glucosinolates (GTP, GNS, GBB). These glucosinolates, which were found in Chinese cabbage seeds, matched up with earlier reports [1].

**Figure 1:**
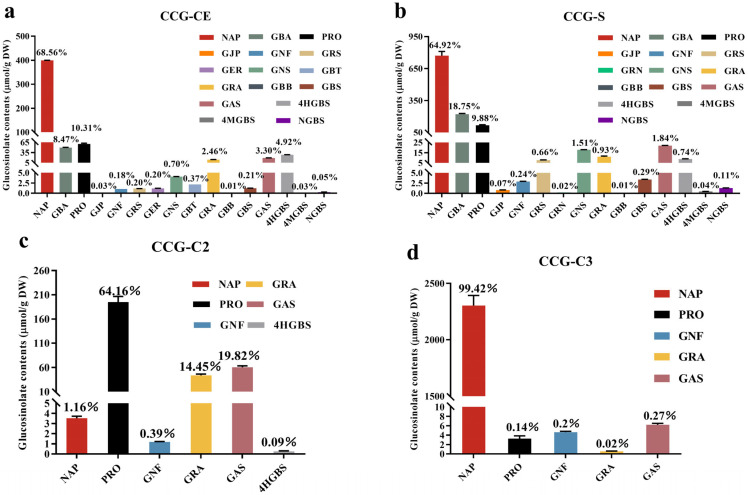

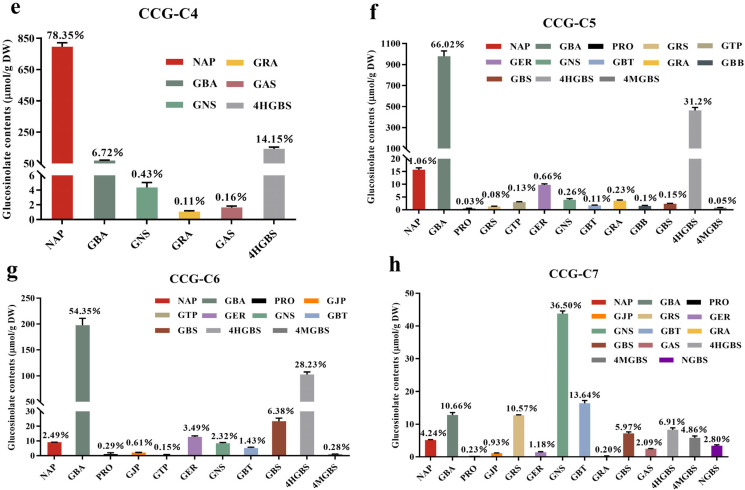
Comparison of the types and contents of glucosinolates of crude extracts (**a**), collections by acidic alumina column chromatography (**b**), and collection fractions (collection 2–7) by preparative reversed-phase liquid chromatography (**c**–**h**). The seventeen individual glucosinolates included gluconapin (NAP), glucobrassicanapin (GBA), progoitrin (PRO), glucojiaputin (GJP), gluconapoleiferin (GNF), glucoraphasativusain (GRS), glucotropaeolin (GTP), glucoerucin (GER), gluconasturtiin (GNS), glucoberteroin (GBT), glucoraphanin (GRA), glucobarbarin (GBB), glucobrassicin (GBS), glucoalyssin (GAS), hydroxyglucobrassicin (4HGBS), 4-methoxyglucobrassicin (4MGBS), and neoglucobrassicin (NGBS). The percentage marked directly above the bar chart represents the purity of the corresponding glucoside. Data are presented as the mean ± SD (n = 3).

As shown in Figure 1, the main glucosinolates in Chinese cabbage seeds were NAP, GBA, and PRO, with NAP having the highest percentage (68.56%), which is consistent with previous studies [14]. Our earlier research showed that, in comparison to the seeds, the glucosinolate profiles dropped after germination, which may contribute to the dilution of glucosinolate contents during tissue expansion [15]. As a result of isolating and purifying the glucosinolate in the seeds, a certain amount of high-purity glucosinolate samples can be acquired, making it easier to screen for glucosinolate species with anticancer activity [21]. GER and GBT were not detected in the CCG-S fraction compared with the crude GSL extracts, which was likely due to the process of removing the salt ion (KNO_3_).

Further observing the glucosinolate proportions of CCG-(C1-C7) collected by preparative liquid chromatography from Figure 1c–h, different GSL types and compositions were found among the collected fractions. Fraction CCG-C1 did not contain any GSL. The main GSLs in CCG-C2 were PRO, GAS, and GRA, with the highest percentage of 64.16% in the detected GSLs. NAP was the predominant GSL in CCG-C3 and CCG-C4, accounting for 99.42% and 78.35% of the detected GSLs, respectively. The main GSLs in CCG-C5 and CCG-C6 were GBA and 4HGBS. GNS was the main GSL in CCG-C7, accounting for 36.50%. The primary GSLs in CCG-C2 were PRO, GAS, and GRA, accounting for 64.16%, 19.82%, and 14.45% of the detected GSLs. NAP made up the majority of the discovered GSLs in CCG-C3 and CCG-C4, accounting for 99.42% and 78.35%, respectively. GBA and 4HGBS served as the primary GSLs in CCG-C5 and CCG-C6. Additionally, 36.50% of GSL in CCG-C7 came from GNS. Because of the role of glucosinolates in cancer prevention, it was necessary to precisely identify types of antitumor-active GSLs in diverse vegetables. The absence of sufficient quantities of pure glucosinolate reference materials impeded this effort. Smaller amounts of glucoraphanin from broccoli had been prepared using preparative HPLC [23]. In our study, the purity of the fraction CCG-C3 was 99.56%, and it contained more than 99% NAP. As a result, high-purity NAP (>99%) could be produced using preparative liquid chromatography.

### 3.2. Identification of NAC-ITCs

The total ion chromatogram (TIC) is shown in Figure S2. The NAC-ITC conjugates were isolated using UHPLC based on retention time and identified using UHPLC-Q-TOF-MS. The potential chemical composition of NAC-ITC conjugates was deduced based on the mass-to-charge ratio of the primary mass spectrometry molecular ions and secondary mass spectrometry fragment ions, and the structures were confirmed with recently published articles [18,19]. NAC-ITCs were detected in positive ionization (PI) modes, with a prominent molecular ion peak [M + H]^+^ defining fragmentation patterns. Sodium adducts [M + Na]^+^ and *N*-acetyl-L-cysteine [NAC + H]^+^ (*m*/*z* 164) were the other most abundant fragment ions for NAC-ITCs in PI mode [19]. Therefore, by detecting the characteristic product ions of neutral loss of fragments and fragment ions created from side chain groups produced by TOF-MS-MS, the identification of NAC-ITCs could be completed.

**Figure 2:**
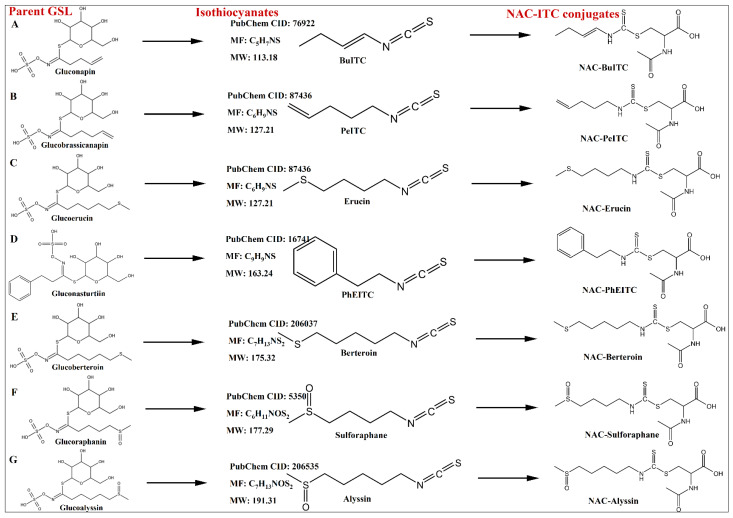
Structures of the identified isothiocyanates, the parent glucosinolates, and NAC-ITC conjugates. PubChem CID, PubChem Identifier from the database of chemicals; MW, molecular weight (g/mol); MF, molecular formula.

The mass spectrometry data of NAC-ITCs is shown in Table 2. The structures of the identified isothiocyanates, the parent glucosinolates, and NAC-ITC conjugates are shown in Figure 2. A total of seven NAC-ITCs were identified according to the spectrometry cracking characteristics of NAC-ITCs. For example, NAC-3-butenyl ITC (NAC-BuITC) with [M + H]^+^ at *m*/*z* 277, fragmented mainly to *m*/*z* 299 and *m*/*z* 164 due to sodium adducts [M + Na]^+^ and *N*-acetyl-L-cysteine [NAC + H]^+^. NAC-Erucin, NAC-Sulforaphane, and NAC-Alyssin mainly fragmented sodium adducts [M + Na]^+^. Among the detected NAC-ITCs, six of the parent GSLs belonged to aliphatic glucosinolates (NAP, GBA, GER, GBT, GRA, and GAS) and one was aromatic glucosinolates (GNS). On the other hand, the NAC-ITC derivatives of indolic glucosinolates were not detected.

Given the volatility of ITCs, gas chromatography (GC) has been frequently used for ITC analysis [17]. Several ITCs in the GC injection port, however, were thermally unstable [24]. Previously, liquid chromatography (LC) analysis of ITCs was established using derivatization with thiol compounds, which could overcome the limitations of GC analysis [18,19]. An LC-MS approach for quantifying several individual ITCs was specifically derivatized with *N*-acetyl-L-cysteine (NAC). This derivatization enabled the ionization of ITCs while preserving their R-groups [19]). The derivatization process of ITC with NAC is shown in Figure S3B [18].

In our study, ITCs were the default rearrangement products of aglycones in GSL extracts and purifications treated with commercially available myrosinase, which were in accordance with earlier reports [18,25]. As shown in Figure 1a, NAP, GBA, and PRO were abundant in Chinese cabbage seeds. NAC-BuITC and PeITC, derivatives of NAP and GBA, were identified in crude extracts and purified fractions containing these two glucosinolates, respectively. The products of a mixture of PRO (2-hydroxy-3-butenyl GSL), myrosinase, and NAC were unknown. After being treated with myrosinase, PRO produced unstable 2-OH-3-butenyl ITC, which spontaneously cyclized to generate 5-ethenyl-1,3-oxazolidine-2-thione (i.e., goitrin) [26]. There has been little literature available on whether goitrin or the unstable 2-OH-3-butenyl ITC had reacted with NAC. Andini et al. [18] pointed out that the hydrolysis product of PRO and NAC might generate a dimer of the reaction product with a molecular formula of C_20_H_32_N_4_O_8_S_4_. Similarly, after being treated with myrosinase, indolic GSLs degrade into unstable ITCs, which then degrade into other products [27]. For example, 3-indolylmethyl GSL (GBS) produced several hydrolysis products, such as indole-3-acetonitrile and 3-indolylmethyl ITC, which then combined with water to generate indole-3-carbinol [27,28,29]. However, neither these molecules nor any potential byproducts of their reactions with NAC were found, which was consistent with previous studies [18].

GSL’s biological activity was determined by how it was hydrolyzed or broken down, as well as the hydrolysis products [2]. The process of generating ITC from GSL hydrolysis by myrosinase was shown in Figure S3A [18]. Due to the high SFN concentration of cooked broccoli, the analysis of GSL hydrolysis products was primarily concentrated on this vegetable [17,24,25]. Contrarily, there were fewer pertinent investigations on Chinese cabbage because Chinese cabbage only contains small amounts of the already-recognized GSL types having anticancer action. As a result, it is crucial to research the types of GSL hydrolysis products found in Chinese cabbage seeds and their anticancer activity.

### 3.3. Antitumor Activity of ITCs on Human Cancer Cells

**Figure 3:**
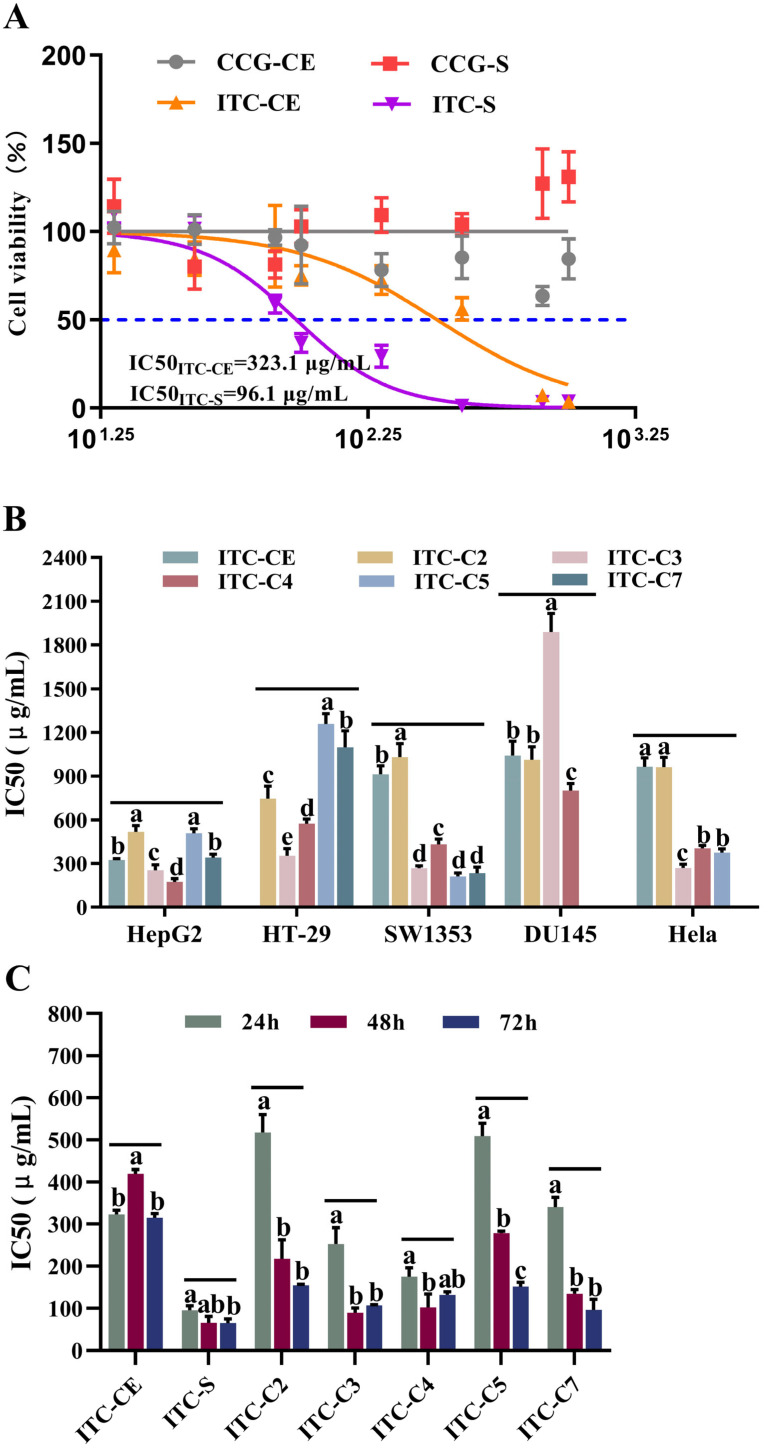
The anticancer activity of glucosinolates and isothiocyanates in vitro. (**A**) The effect of different concentrations of CCG-CE, CCG-S, ITC-CE, and ITC-S on HepG2 cell viabilities. (**B**) IC50 values of ITC-CE and ITC-(C2-C7) on different tumor cell lines for 24 h. (**C**) IC50 values of ITC-CE, ITC-S, and ITC-(C2-C7) on HepG2 cells for 24 h, 48 h, and 72 h. Different lowercase letters (a, b, c, d, and e) indicated significant differences (n = 6, one-way ANOVA; LSD and Tamhane T2’s tests; *p* < 0.05).

MTT assay was used for evaluating the antiproliferation ability of glucosinolates and their hydrolysis products, isothiocyanates, on HepG2 cells, as illustrated in Figure 3A. Fractions ITC-CE and ITC-S significantly reduced the viability of HepG2 cells in a dose-dependent manner when compared with CCG-CE and CCG-S. It suggested that the hydrolysis product of GSLs, isothiocyanates, exhibited antitumor action, which was consistent with earlier reports [1,2].

Since the antitumor activity of ITC was elucidated, the inhibitory effects of ITC-CE and purified fractions ITC-(C1-C7) on different tumor cells were further investigated. The cell survival rates of ITCs on five different types of tumor cells were measured: human liver cancer cells (HepG2), human colorectal adenocarcinoma cells (HT-29), human osteosarcoma cells (SW1353), human prostate cancer cells (DU145), and human cervical cancer cells (Hela). ITC-C1 and ITC-C6 demonstrated no inhibitory effect on target tumor cells based on IC50 values. The absence and lower purity of GSLs in CCG-C1 (3.56 ± 0.14%) and CCG-C6 (21.47 ± 3.21%), the fraction before enzymatic digestion, could explain the the lack of anticancer activity. As shown in Figure 3B, due to the low GSL content in CCG-CE (32.12 ± 4.29%), ITC-CE inhibited HepG2, SW1353, DU145, and Hela cells at higher doses, with IC50 values of 323.1 μg/mL, 917.1 μg/mL, 1066.0 μg/mL, and 963.8 μg/mL, respectively. The fractions ITC-C2, ITC-C3, ITC-C4, ITC-C5, and ITC-C7 showed different inhibitory abilities against tumor cells. For example, ITC-C3 showed an inhibitory effect on HepG2 cells, HT-29 cells, SW1353 cells, and Hela cells with IC50 values ranging from 253.13 to 353.93 μg/mL. In contrast, ITC-C2, ITC-C4, ITC-C5, and ITC-C7 demonstrated certain differences in inhibitory efficacy against tumor cells. For example, the IC50 value of ITC-C5 on the survival of SW1353 cells was 210.90 μg/mL but had no inhibitory effect on DU145 cells. The results demonstrated that BuITC, the enzymatic product of NAP, exerted cytotoxic effects and inhibited the growth of HepG2, HT-29, SW1353, and Hela cells.

**Figure 4:**
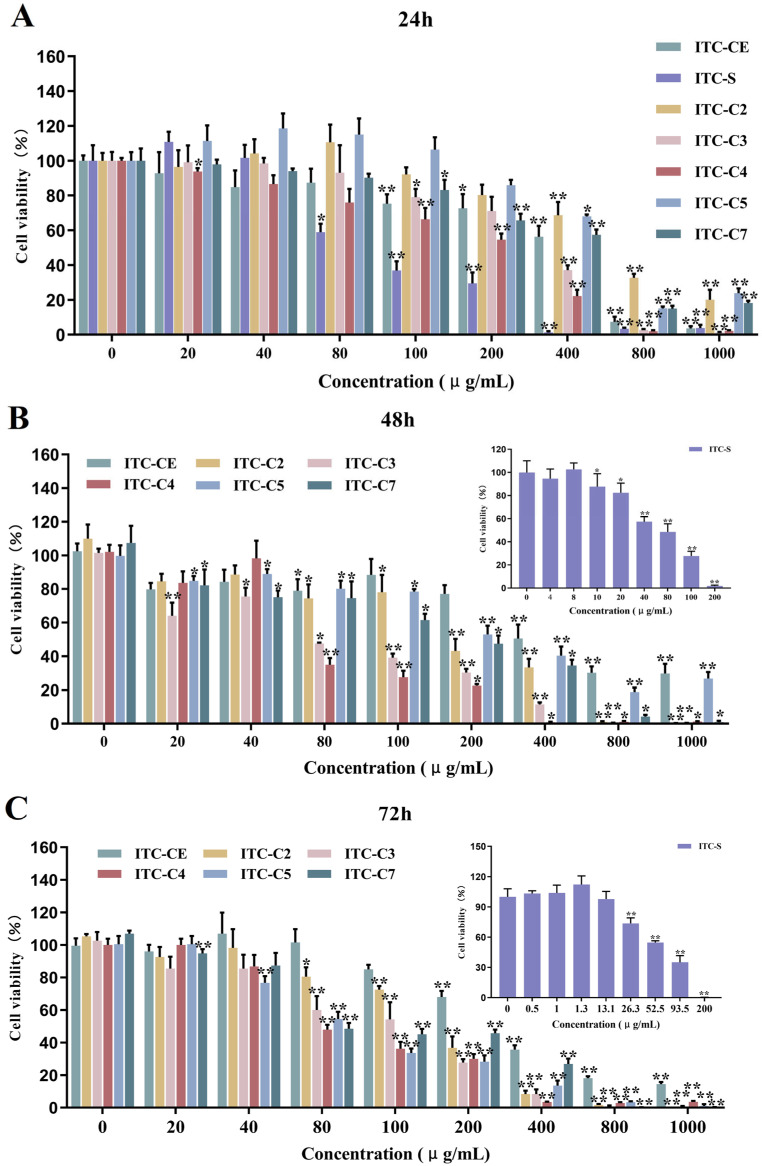
The cell viabilities of different concentrations of ITC-CE and ITC-(C2-C7) on HepG2 cell viabilities for 24 h (**A**), 48 h (**B**), and 72 h (**C**). ‘*’ (*p* < 0.05) and ‘**’ (*p* < 0.01) indicated significant differences compared with the normal control group (n = 6, one-way ANOVA; LSD and Tamhane T2’s tests).

According to the inhibitory effects of ITCs on tumor cells, HepG2 cells were chosen in the following investigations. The time effect of fractions (ITC-CE, ITC-S, ITC-C2, ITC-C3, ITC-C4, ITC-C5, and ITC-C7) on the inhibition of HepG2 cells was further explored based on IC50 values and cell viabilities when HepG2 cells were treated with different concentrations of samples for 24 h, 48 h, and 72 h. As shown in Figure 3C, the longer the administration time, the lower the IC50 values. The IC50 values for 48 h and 72 h were significantly lower than those for 24 h (*p* < 0.05), indicating that prolonging the treatment time significantly improved the inhibitory ability of ITC on HepG2 cells (*p* < 0.05). When the cells were treated with ITC-C3 for 24 h and 48 h, the IC50 values reduced from 253.13 μg/mL to 89.44 μg/mL. In addition, fraction ITC-S exhibited a strong inhibitory effect on HepG2 cells after treatment for 24 h (95.96 μg/mL), and treatment duration had a better effect on the IC50 values, which decreased from 95.96 μg/mL to 64.98 μg/mL. This is because the fraction CCG-S had a higher purity and more GSLs types, so it contained more types of ITCs after degradation by exogenous myrosinase, and it could achieve a better inhibitory effect at 24 h of treatment of HepG2 cells, implying that different types of ITCs have a synergistic effect on the inhibition of HepG2 cells. Based on the types, purities, and percentages of glucosinolate and isothiocyanate in different fractions before and after enzymatic digestion, we deduced several isothiocyanates with inhibitory effects on HepG2 cells, namely, SFN in ITC-C2, BuITC in ITC-C3 and ITC-C4, and PhEITC in ITC-C7, which is consistent with other studies [3,10,13]. The viabilities of HepG2 cells under different administration time treatments are shown in Figure 4. The cell viabilities were dose-dependent on the sample concentration. The cell viability declined as ITC concentration increased.

Numerous epidemiological studies have demonstrated a high correlation between the consumption of cruciferous vegetables rich in ITCs and a low cancer risk [3]. Sulforaphane (SFN), phenethyl isothiocyanate (PEITC), benzyl isothiocyanate (BITC), and allyl isothiocyanate (AITC) have antitumor activities [10,30]. Research indicated that SFN inhibited tumor development by affecting multiple molecular targets within oncogenic pathways [3]. Studies have demonstrated that at concentrations exceeding 20 µM, SFN significantly reduced the survival rate of HepG2 cells, induced DNA strand breaks, and suppressed the tumorigenicity of HepG2 cells [31]. In addition, SFN exhibited antioxidant capabilities. While inducing cell cycle arrest and apoptosis in HeLa cells, SFN concurrently increased nitric oxide (NO) levels [32]. Furthermore, SFN inhibited the proliferation of DU145 by mediating the upregulation of ERK1/2 phosphorylation, coupled with an increase in anti-migration gene expression and a decrease in pro-migration gene expression [33]. In our study, ITC-C2 showed varying degrees of inhibition on tumor cells after being treated for 24 h. In particular, the IC50 values for ITC-C2 dropped from 517.8 to 154.35 μg/mL after acting on HepG2 cells for 72 h. We assumed that SFN might be the active component in ITC-C2 based on the distribution of GSLs and ITCs in CCG-C2 and ITC-C2 [3]. A similar active site was found in fraction ITC-C7. When the HepG2 cells were treated with ITC-C7 for 72 h, the IC50 value was 96.38 μg/mL, which decreased by 71.70% compared with 24 h of treatment. However, due to its low purity, it was challenging to determine which component was in the active site. Given that GNS made up roughly 36.5% of CCG-C7 and that PhEITC, the degradation product of GNS, had a higher anticancer activity, it was assumed that PhEITC had antitumor activity in fraction ITC-C7 [11]. As the precursor glucosinolate of PhEITC, GNS is relatively abundant in Brussels sprouts, broccoli, cabbage, and cauliflower [3]. PhEITC demonstrated inhibitory effects against multiple cancer cell lines, including MCF-7 breast cancer cells (IC_50_ = 5.02 μM), HeLa cervical cancer cells (10 μM), HepG2 liver cancer cells (IC_50_ = 7.83 μM), and DU145 prostate cancer cells (10 μM) [3]. The inhibitory concentrations varied considerably across different tumor cell types. PhEITC exerted its anticancer effects primarily by activating phase II enzymes and antioxidant enzymes and enhancing detoxification mechanisms which suppressed the proliferation, invasion, and migration of tumor cells [34]. Based on the above reports, our findings also implied that SFN and PhEITC might exert an antitumor action at low concentrations, which may be helpful in epidemiological studies about the link between cruciferous vegetable consumption and the risk of human malignant diseases. Previous research demonstrated that BuITC had better antiproliferative efficacy against PC-3 cells in prostate cancer cell lines, but not against DU145 cells, which was consistent with our results [13]. Further studies are required to verify the inhibitory effect of BuITC on PC-3 cells.

### 3.4. Effects of ITC-C3 on the Apoptosis of HepG2 Cells

**Figure 5:**
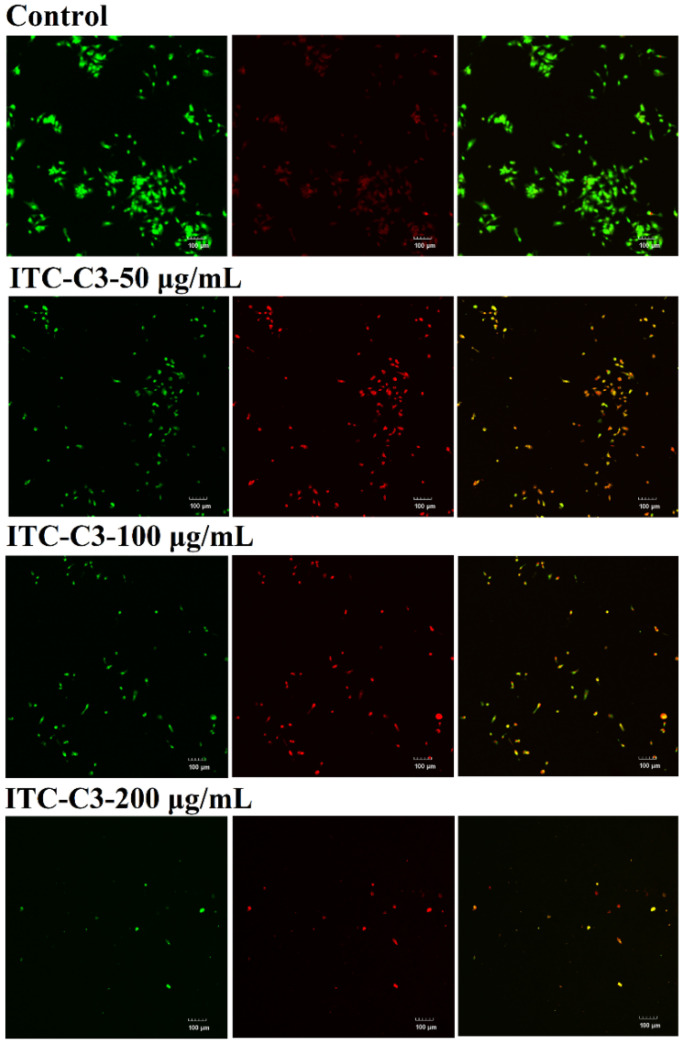
The apoptotic effects of ITC-C3 at different concentrations on HepG2 cells were observed using fluorescence microscopy with AO/EB staining.

Given CCG-C3′s high purity, the major component of its hydrolysis product was BuITC, hence fraction ITC-C3 was selected as the sample for treating HepG2 cells to further explore the inhibition mechanism. AO/EB staining further verified the apoptosis and necrosis in different stages of HepG2 cells. Acridine Orange (AO) could freely penetrate intact cell membranes and bind to DNA in the nucleus, exciting the production of green fluorescence. Ethidium Bromide (EB) could not penetrate cells with intact membranes. However, it could enter cells with damaged membranes and increased permeability, where it intercalated into nuclear DNA and emited orange fluorescence [35]. As shown in Figure 5, HepG2 cells from the control group showed green fluorescence and normal morphologies. After treatment with 50 μg/mL of fraction ITC-C3 for 48 h, the green fluorescence became weaker and the red fluorescence increased, indicating an increase in cell membrane permeability and significant apoptotic features of the cells. This biological process showed a dose-dependent manner. The fluorescence was dramatically diminished when the sample concentration was 200 μg/mL, and it was assumed that the majority of the HepG2 cells underwent apoptosis and were washed out by PBS during the staining process.

**Figure 6:**
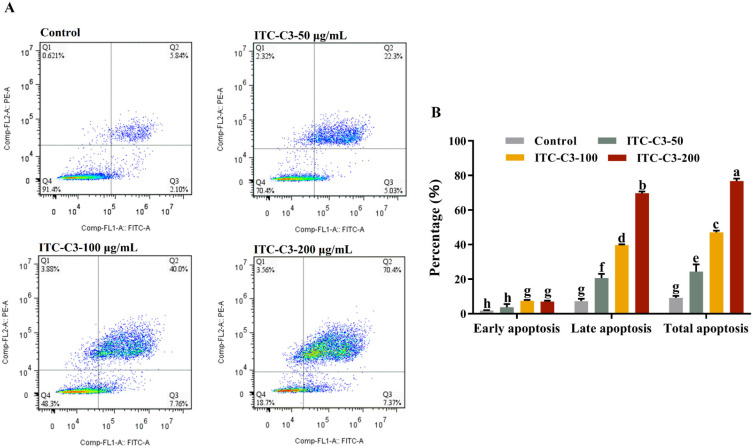
The status of apoptotic cells (**A**) and apoptosis rate (**B**) were observed using a flow cytometer. Different lowercase letters (a, b, c, d, e, f, g, and h) indicated significant differences (n = 3, one-way ANOVA; LSD and Tamhane T2’s tests; *p* < 0.05).

To distinguish living and apoptotic cells, flow cytometry analysis using Annexin V-FITC/PI double staining was also carried out. As shown in Figure 6A, the apoptosis of HepG2 cells increased steadily after treatment with ITC-C3 for 48 h. The apoptosis rates were dose-dependent on the concentrations of ITC-C3, which was consistent with the MTT results. The proportions of living cells in the three different concentrations (50 μg/mL, 100 μg/mL, and 200 μg/mL) of ITC-C3-treated groups reduced from 91.4% to 70.4%, 48.3%, and 18.7% in the control group, demonstrating that BuITC suppressed cell growth and promoted apoptosis. The total apoptosis rates of HepG2 cells were significantly higher in all ITC-C3-treated groups compared with the normal control group (*p* ˂ 0.05). The total apoptosis rates of the HepG2 cells induced by three different concentrations of ITC-C3 were 27.33%, 47.76%, and 77.77%, respectively. Compared with the normal control, the proportion of early and late apoptotic cells treated with different concentrations of ITC-C3 increased from 2.10% to 7.37% (early apoptotic cells) and 5.84% to 70.4% (late apoptotic cells), respectively. The results suggested that BuITC tended to induce cell death through cell apoptosis in HepG2 cells.

Few studies related to the inhibition of tumor cells by BuITC have been reported. The mechanism of the inhibitory effect of BuITC on tumor cells is not fully understood. Other ITCs, including SFN, PhEITC, BITC, and AITC had shown antiproliferative, pro-apoptotic, anti-migratory, anti-inflammatory, and anti-angiogenic effects against several cancers [3]. Certain ITCs could act in all stages of carcinogenesis. SFN and PhEITC could affect the conversion of carcinogens during the early stages of carcinogenesis by controlling the CYP enzymes responsible for their metabolisms. By activating phase II CYP proteins, carcinogens were eliminated or converted into non-toxic metabolites [36]. Inflammation was a major contributor to carcinogenesis and the inhibition of inflammatory pathways, particularly the NF-κB pathway, might lower the risk of cancer. BITC and SFN had been proven in studies to reduce inflammation by modifying inflammatory vesicle activity by lowering NF-κB expression and nuclear localization [36,37]. Furthermore, BITC could induce the apoptosis of tumor cells by altering their redox status, specifically by inhibiting the expression of SOD and GSH, increasing oxidative stress, which in turn induced apoptosis in tumor cells [38]. Furthermore, SFN modulated various cellular signaling pathways (PI3K, Wnt, MAPK, and Hedgehog) as well as critical regulators of tumor growth, invasion, migration, cell survival, and angiogenesis [39]. Epigenetically, SFN, PhEITC, BITC, and AITC further enhanced their transcriptional regulation of key cellular regulators by modulating the expression and activity of DNA methyltransferases, histone modifiers, and miRNAs, allowing ITC to exercise its anticancer potential [12,37,40,41]. In our study, BuITC induced the apoptosis of HepG2 cells but did not increase the generation of ROS. In conjunction with the pro-tumor cell apoptosis mechanisms of other ITCs, changes in key regulators associated with inflammatory pathways, cell cycle arrest, cell migration and invasion, angiogenesis, and epigenetics should be investigated further to probe the mechanism of BuITC’s promotion of HepG2 cell apoptosis.

## 4. Conclusions

In this study, GSL-rich fractions (CCG-S and CCG-C_1-7_) were isolated and purified using acidic Al_2_O_3_ column chromatography and preparative HPLC, from which high-purity NAP (>99%) was obtained. The profiles of GSLs and their enzymatic products, ITCs, were identified using UHPLC-Q-TOF-MS. Seventeen glucosinolates were identified, and NAP, GBA, and PRO were the dominant GSLs in Chinese cabbage seeds. Among the detected ITCs, six of the parent GSLs belonged to aliphatic glucosinolates and one was aromatic glucosinolates. BuITC, SFN, and PhEITC showed potential antiproliferative activity against HepG2 cells and had synergistic-, dose-, and time-dependent effects. Moreover, BuITC, the parent GSL NAP rich in Chinese cabbage, tended to induce cell death through cell apoptosis in HepG2 cells. The changes in key regulators associated with inflammatory pathways, cell cycle arrest, cell migration and invasion, angiogenesis, and epigenetics should be further investigated. In addition, future research should be targeted at the application of molecular breeding to increase beneficial glucosinolate levels.

## 5. Chemical Compounds Studied in This Article

Gluconapin (PubChem CID: 9548620); Glucobrassicanapin (PubChem CID: 5485207); Progoitrin (PubChem CID: 5281139); Glucojiaputin (PubChem CID: 46173879); Gluconapoleiferin (PubChem CID: 5281136); Glucoraphasativusain (PubChem CID: 122220424); Glucotropaeolin (PubChem CID: 656498); Glucoerucin (PubChem CID: 656539); Gluconasturtiin (PubChem CID: 5464032); Glucoberteroin (PubChem CID: 656525); Glucoraphanin (PubChem CID: 9548634); Glucobarbarin (PubChem CID: 46173883); Glucobrassicin (PubChem CID: 6602378); Glucoalyssin (PubChem CID: 9589398); 4-Hydroxyglucobrassicin (PubChem CID: 656561); 4-Methoxyglucobrassicin (PubChem CID: 656563); Neoglucobrassicin (PubChem CID: 9576416); 3-butenyl ITC (PubChem CID: 76922); PeITC: 4-pentenyl ITC (PubChem CID: 87436); Erucin: 4-(methylthio) butyl ITC (PubChem CID: 78160); PhEITC: (2-phenylethyl ITC) (PubChem CID: 16741); Berteroin: 4-(methylsulfinyl)-3-butenyl ITC (PubChem CID: 206037); Sulforaphane: 4-(methylsulfinyl) butyl ITC (PubChem CID: 5350); Alyssin: 5-methylsulfinylpentyl ITC (PubChem CID: 206535).

## Figures and Tables

**Table 1:** Glucosinolate compounds analyzed in Chinese cabbage seeds and their mass spectrometry results.

Compounds	Chemical Formula	Precursor Ions, *m*/*z*	Retention Time/min	Mass Error/ppm	Fragments, *m*/*z*
Gluconapin (NAP)	C_11_H_19_NO_9_S_2_	372.0425	1.56	−0.9	372.04, 274.99, 259.01, 241.00, 96.96
Glucobrassicanapin (GBA)	C_12_H_21_NO_9_S_2_	386.0586	2.7	0.2	386.05, 274.99, 259.01, 241.00, 96.96
Progoitrin (PRO)	C_11_H_19_NO_10_S_2_	388.0374	0.97	−0.9	388.03, 274.99, 259.01, 241.00, 96.96
Glucojiaputin (GJP)	C_12_H_23_NO_9_S_2_	388.073	2.99	−3	388.07, 259.01, 96.96
Gluconapoleiferin (GNF)	C_12_H_21_NO_10_S_2_	402.0533	1.17	−0.2	402.05, 274.99, 259.01, 96.96
Glucoraphasativusain (GRS)	C_13_H_25_NO_9_S_2_	402.0894	4.20	−1.1	402.09, 259.01, 96.96
Glucotropaeolin (GTP)	C_14_H_19_NO_9_S_2_	408.041	2.24	−4.5	408.04, 96.96
Glucoerucin (GER)	C_12_H_23_NO_9_S_3_	420.0469	2.93	1.5	420.05, 96.96
Gluconasturtiin (GNS)	C_15_H_21_NO_9_S_2_	422.0583	3.78	−0.6	422.05, 274.99, 259.01, 96.96
Glucoberteroin (GBT)	C_13_H_25_NO_9_S_2_	434.0615	3.74	−0.8	434.06, 274.99, 259.01, 96.95
Glucoraphanin (GRA)	C_12_H_23_NO_10_S_3_	436.0409	0.92	−0.5	436.04, 421.02, 388.04, 372.04, 178.01
Glucobarbarin (GBB)	C_15_H_21_NO_10_S_2_	438.0533	3.38	−0.2	438.05, 274.99, 96.96
Glucobrassicin (GBS)	C_16_H_20_N_2_O_9_S_2_	447.0537	3.35	−0.1	447.05, 274.99, 259.01, 96.96
Glucoalyssin (GAS)	C_13_H_25_NO_10_S_3_	450.0564	1.06	−0.7	450.06, 386.05, 274.99, 259.01, 241.00, 96.96
4-Hydroxyglucobrassicin (4HGBS)	C_16_H_20_N_2_O_10_S_2_	463.0485	2.21	−0.3	463.05, 274.99, 259.01, 241.00, 96.95
4-Methoxyglucobrassicin (4MGBS)	C_17_H_22_N_2_O_10_S_2_	477.0644	3.98	0.2	477.06, 96.96
Neoglucobrassicin (NGBS)	C_17_H_22_N_2_O_10_S_2_	477.0644	4.62	0.2	477.06, 446.04, 96.96

**Table 2:** Mass spectrometry results of isothiocyanates compounds (analyzed as NAC-ITC conjugates) using UHPLC-Q-TOF-MS.

NAC-ITC Conjugates	Isothiocyanates			Chemical Formula	Precursor Ions, *m*/*z*	Retention Time/min	Mass Error/10^−6^	Fragments, *m*/*z*	Detected Fractions
	Chemical Name	Mw	Parent GSL						
NAC-BuITC	BuITC	113.18	NAP	C_10_H_16_N_2_O_3_S_2_	277.0675	29.68	−0.1	299.0487, 277.0675, 164.0366, 130.0494, 122.0262, 76.0213	ITC-CE, ITC-S, ITC-C3, ITC-C4, ITC-C5, ITCC6, ITC-C7
NAC-PeITC	PeITC	127.21	GBA	C_11_H_18_N_2_O_3_S_2_	291.0828	34.53	−1.4	313.0646, 291.0828, 164.0372, 130.0495, 122.0266, 76.0208	ITC-CE, ITC-S, ITC-C4, ITC-C5, ITC-C6, ITC-C7
NAC-Erucin	Erucin	161.29	GER	C_11_H_20_N_2_O_3_S_3_	325.0704	34.27	−1.6	347.0518, 325.0704, 130.0495,	ITC-C4, ITC-C5, ITC-C6
NAC-PhEITC	PhEITC	163.24	GNS	C_14_H_18_N_2_O_3_S_2_	327.0824	38.08	−2.4	349.0643, 327.0824, 164.0374, 130.0647, 122.0265, 76.0209	ITC-S, ITC-C4, ITC-C5, ITC-C6, ITC-C7
NAC-Berteroin	Berteroin	175.32	GBT	C_12_H_22_N_2_O_3_S_3_	339.086	37.86	−1.7	361.0675, 339.0860, 164.0373, 130.0495, 122.0266, 76.0209	ITC-CE, ITC-C4, ITC-C5, ITC-C6, ITC-C7
NAC-Sulforaphane	Sulforaphane	177.29	GRA	C_11_H_20_N_2_O_4_S_3_	341.0651	16.15	−2.0	363.0464, 341.0651	ITC-CE, ITC-S, ITC-C2, ITC-C4, ITC-C5, ITC-C6
NAC-Alyssin	Alyssin	191.31	GAS	C_12_H_22_N_2_O_4_S_3_	355.0812	21.92	−0.8	377.0625, 355.0812	ITC-CE, ITC-S, ITC-C2, ITC-C3, ITC-C4, ITC-C5, ITC-C6, ITC-C7

Isothiocyanates abbreviations: ITC—isothiocyanates; BuITC—3-butenyl ITC; PeITC—4-pentenyl ITC; Erucin—4-(methylthio) butyl ITC; PhEITC—(2-phenylethyl ITC); Berteroin—4-(methylsulfinyl)-3-butenyl ITC; Sulforaphane—4-(methylsulfinyl) butyl ITC; Alyssin—5-methylsulfinylpentyl ITC. Glucosinolate abbreviations: GSL—glucosinolate; NAP—gluconapin (3-butenyl GSL); GBA—glucobrassicanapin (4-pentenyl GSL); GER—glucoerucin (4-methylthiobutyl GSL); GNS—gluconasturtiin (2-phenylethyl GSL); GBT—glucoberteroin (5-methylthiopentyl GSL); GRA—glucoraphanin (4-methylsulfinylbutyl GSL); GAS—glucoalyssin (5-methylsulfinylpentyl GSL).

## Data Availability

The original contributions presented in the study are included in the article/Supplementary Material, further inquiries can be directed to the corresponding author/s.

## Supplementary Materials

The following supporting information can be downloaded at: https://www.mdpi.com/article/10.3390/foods14162808/s1, Figure S1: The elution curves of crude glucosinolate extracts by preparative liquid chromatography (A) and the purities of the glucosinolate fractions (B); Figure S2: The total ion chromatogram (TIC) corresponding to the analysis of NAC-ITC conjugates by UHPLC/TOF-MS/MS; Figure S3: (A) The process of generating ITC from GSL breakdown by myrosinase at pH 5-7. (B) The derivatization process of ITC with NAC.

## Abbreviations

GSL, glucosinolate; HPLC, high-performance liquid chromatography; ITC, isothiocyanate; UHPLC-Q-TOF-MS, ultrahigh-performance liquid chromatography–quadrupole time of flight-mass spectrometry; ESI, electrospray ionization source; SIN, sinigrin (2-propenyl GSL); NAP, gluconapin (3-butenyl GSL); GBA, glucobrassicanapin (4-pentenyl GSL); PRO, progoitrin (2-hydroxy-3-butenyl GSL); GJP, glucojiaputin (2-methylbutyl GSL); GNF, gluconapoleiferin (2-hydroxy-4-pentenyl GSL); GRS, glucoraphasativusain (3-methylpentyl GSL); GTP, glucotropaeolin (Benzyl GSL); GER, glucoerucin (4-methylthiobutyl GSL); GNS, gluconasturtiin (2-phenylethyl GSL); GBT, glucoberteroin (4-methylsulfinyl-3-butenyl GSL); GRA, glucoraphanin (4-methylsulfinylbutyl GSL); GBB, glucobarbarin (2-hydroxy-2-phenylethyl GSL); GBS, glucobrassicin (3-indolylmethyl GSL); GAS, glucoalyssin (5-methylsulfinylpentyl GSL); 4HGBS, 4-hydroxyglucobrassicin (4-hydroxy-3-indolylmethyl GSL); 4MGBS, 4-methoxyglucobrassicin (4-methoxy-3-indolylmethyl GSL); NGBS, neoglucobrassicin (1-methoxy-3-indolylmethyl GSL); BuITC, 3-butenyl ITC; PeITC, 4-pentenyl ITC; Erucin, 4-(methylthio) butyl ITC; PhEITC, (2-phenylethyl ITC); Berteroin, 4-(methylsulfinyl)-3-butenyl ITC; SFN, sulforaphane (4-(methylsulfinyl) butyl ITC); Alyssin, 5-methylsulfinylpentyl ITC; HepG2, hepatocellular carcinoma cell line; HT-29, human colon carcinoma cell line, SW1353, human osteosarcoma cell line; DU145, human prostate cancer cell line; Hela, human cervical carcinoma cell line; NAC, *N*-acetyl-L-cysteine; MTT, methylthiazolyldiphenyl-tetrazolium bromide; DMSO, dimethyl sulfoxide; AO/EB, acridine orange/ethidium bromide; PI, positive ionization; PC-3, prostate cancer cell line; SOD, superoxide dismutase; GSH, glutathione reductase; ROS, reactive oxygen species.
