# Supplementary material for: Isolation, Identification, and Antitumor Activities of Glucosinolates and Isothiocyanates in Chinese Cabbage Seeds
Source: Foods. 2025 Aug 13;14(16):2808. doi: 10.3390/foods14162808 (PMC12385190; doi:10.3390/foods14162808)
Supplement: Supplementary file 1 [file foods-14-02808-s001.zip › foods-3740241-supplementary.pdf]

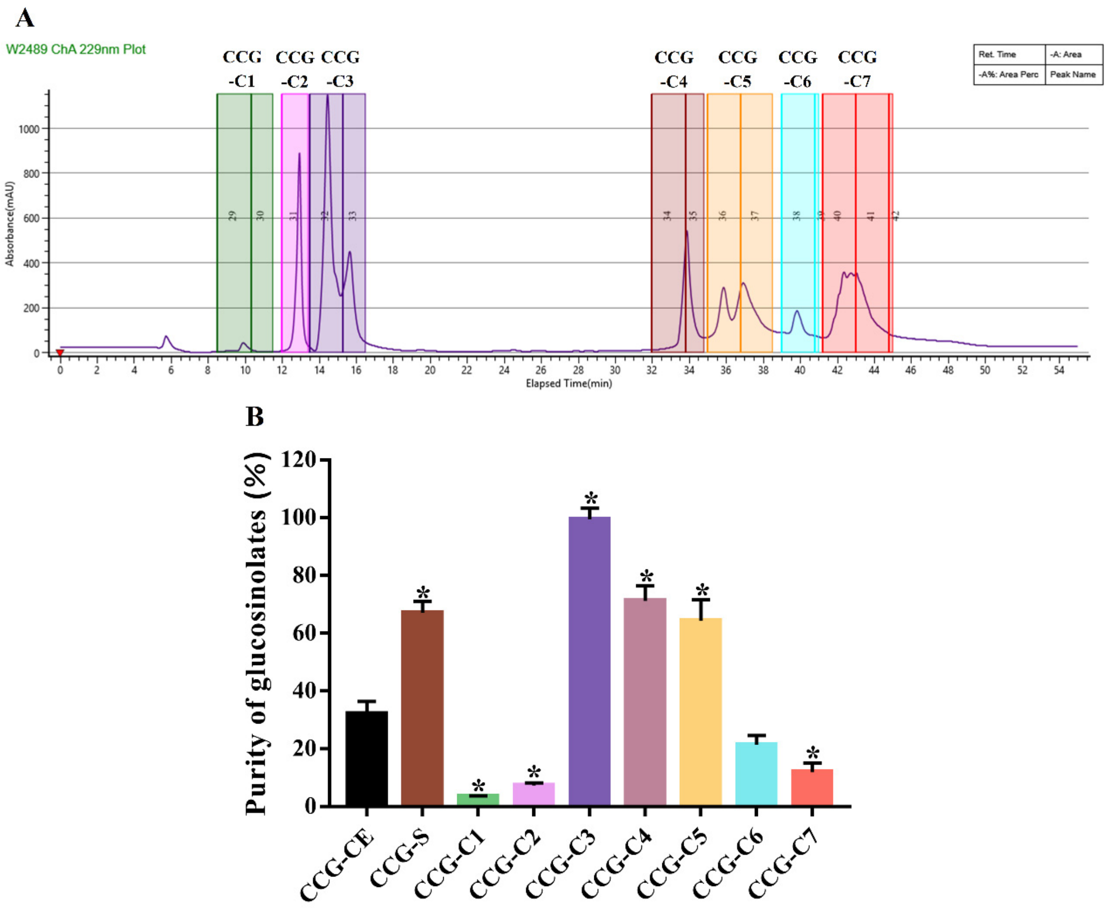

Figure S1 The elution curves of crude glucosinolate extracts by preparative liquid chromatography (A) and the purities of the glucosinolate fractions (B). The fractions were crude extracts from Chinese cabbage seeds (CE), purifiers by acidic alumina chromatography column (P), and collection 1-7 by preparative liquid chromatography (C1-C7), respectively. Data are presented as the mean ± SD (n=3) and analyzed via a one-way ANOVA test followed by LSD and Tamhane T2's tests. "*" indicated significant differences compared to the fraction CCG-CE (*P* < *0.05*).

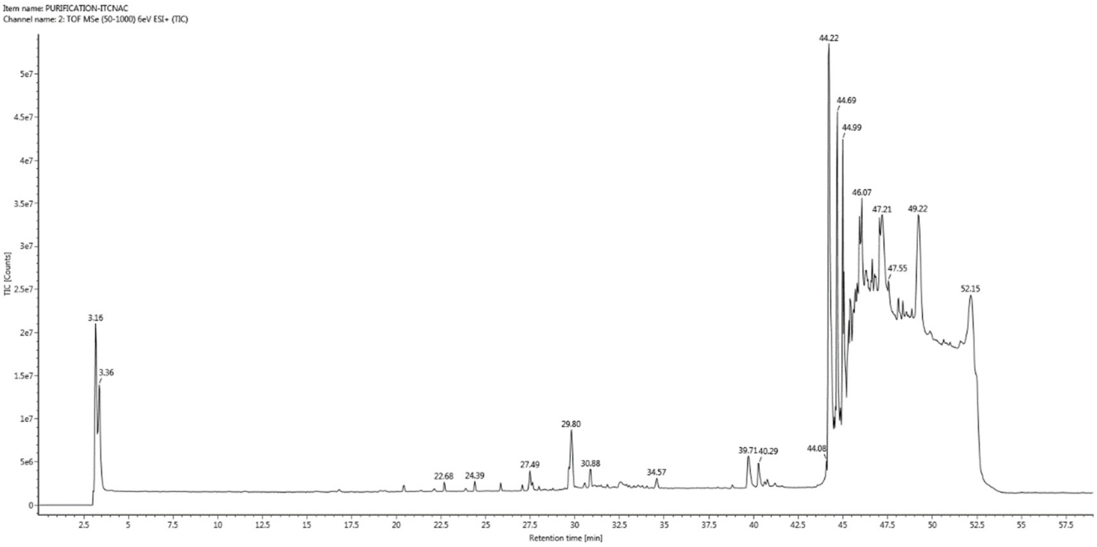

Figure S2 The total ion chromatogram (TIC) corresponding to the analysis of NAC-ITC conjugates by UHPLC/TOF-MS/MS.

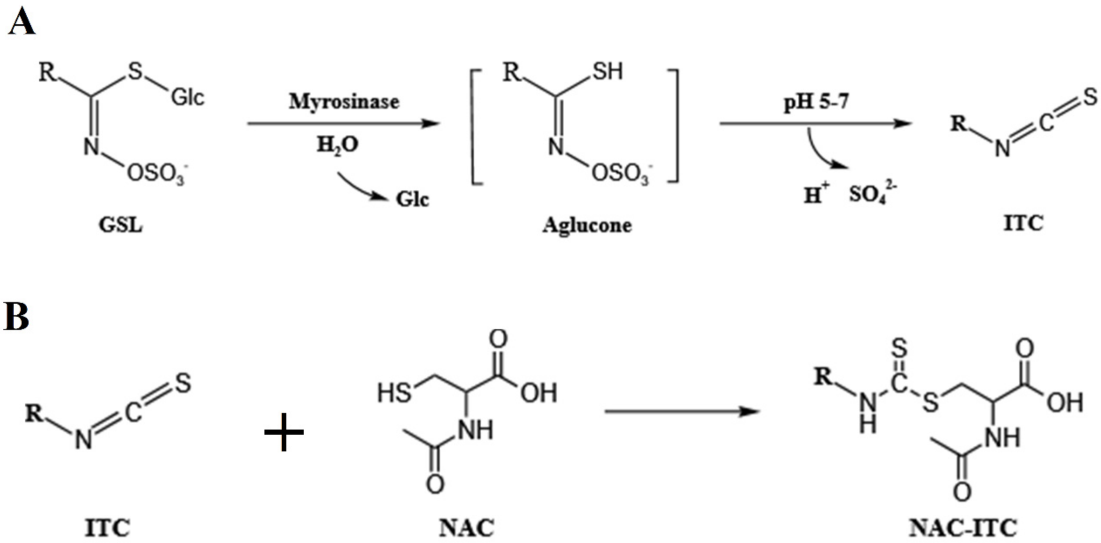

Figure S3 (A) The process of generating ITC from GSL breakdown by myrosinase at pH 5-7. (B) The derivatization process of ITC with NAC (Andini et al., 2020).

References

Andini, S., Araya-Cloutier, C., Sanders, M., & Vincken, J. P. (2020). Simultaneous Analysis of Glucosinolates and Isothiocyanates by Reversed-Phase Ultra-High-Performance Liquid Chromatography-Electron Spray Ionization-Tandem Mass Spectrometry. *Journal of Agricultural and Food Chemistry, 68*(10), 3121-3131. https://doi.org/10.1021/acs.jafc.9b07920.
